# Gender differences in dietary behaviors among Japanese adolescents

**DOI:** 10.1016/j.pmedr.2020.101203

**Published:** 2020-09-11

**Authors:** Yuichiro Otsuka, Yoshitaka Kaneita, Osamu Itani, Maki Jike, Yoneatsu Osaki, Susumu Higuchi, Hideyuki Kanda

**Affiliations:** a Division of Public Health, Department of Social Medicine, Nihon University School of Medicine, Japan; b Division of Environmental and Preventive Medicine, Department of Social Medicine, Faculty of Medicine, Japan; c National Hospital Organization Kurihama Medical and Addiction Center, Japan; d Department of Public Health, Okayama University Graduate School of Medicine Dentistry and Pharmaceutical Sciences, University Faculty of Medicine, Japan

**Keywords:** Adolescents, Dietary behaviors, Cross-sectional study, Gender difference

## Abstract

Unhealthy dietary behaviors in adolescence are an important public health problem. Gender differences in dietary behaviors have already appeared during adolescence. However, few studies have assessed a variety of adolescent dietary behaviors in Japan. We aimed to clarify gender differences in unhealthy dietary behaviors among Japanese adolescents. The participants consisted of 84,988 participants from seventh to 12th grades. Unhealthy dietary behaviors were defined according to the National Health and Nutrition Survey. Multivariable logistic regression was used to analyze a nationally representative sample of Japanese adolescents from the 2014 to 2015 Lifestyle Survey. The effective response rate was 51.4%. The prevalence of unhealthy dietary behaviors (skipping breakfast, snacking, eating out, skipping meals, eating alone at dinner, and subjectively poor diet quality) among boys and girls was 14.2% versus 12.4%, 19.6% versus 14.1%, 10.6% versus 7.0%, 7.9% versus 5.6%, 13.3% versus 12.1%, and 12.3% versus 15.8%, respectively. Compared with boys, girls were more negatively associated with skipping breakfast [OR = 0.76 (95% CI 0.73–0.79)], snacking [OR = 0.67 (95% CI 0.65–0.70)], eating out [OR = 0.62 (95% CI 0.59–0.66)], skipping meals [OR = 0.61 (95% CI 0.58–0.65)], and eating alone at dinner [OR = 0.79 (95% CI 0.76–0.83)]. However, girls were more positively associated with subjectively poor diet quality [OR = 1.19 (95% CI 1.14.1.24)]. The findings suggest that gender differences existed in dietary behaviors. Gender differences in dietary behaviors suggest opportunities for tailoring interventions related to dietary education in schools.

## Highlights

• Few studies assessed a variety of adolescent dietary behaviours in Japan.
• The findings suggest that gender differences existed in dietary behaviors.
• Girls tended to adopt regular dietary behaviors as compared to boys.
• Schools support modeling and reinforcing healthy dietary behaviors.

## 1 Introduction

Adolescence is an important period for physical and psychological development. Dietary behaviors once established in adolescence tend to continue throughout life (Wahl, 1999). Unhealthy dietary behaviors during adolescence can result in nutritional deficiency and delayed growth and may also have a negative effect on performance in school (Kim et al., 2016). Adolescent unhealthy dietary behaviors are associated with other health-related behaviors (Neumark-Sztainer et al., 1997). For example, skipping breakfast has been linked to insomnia (Kaneita et al., 2006). Frequent snacking is a risk factor for poor dietary behaviors, and is related to lifestyle behaviors (Larson et al., 2016). In Korean high school students, distress not only increased instant food and snack intake, but also the frequency of eating out (Hong and Kim, 2014). Skipping meals is an unhealthy method to promote weight loss and a potential risk factor for eating disorders (Neumark-Sztainer et al., 2012). Shared meals may protect against nutritional-related health problems, including obesity, other unhealthy dietary behaviors, and eating disorders (Fulkerson et al., 2014). A cross-sectional study targeting Australian adolescents showed that poor diet quality was associated with depression (Jacka et al., 2010). Furthermore, unhealthy dietary behaviors increase the risk of obesity and non-communicable diseases (Amine et al., 2002, Story et al., 2000). Therefore, unhealthy dietary behaviors in adolescence have been suggested as an important public health issue (Moreno et al., 2014). It is necessary to develop strategies to promote healthier dietary behaviors.

Gender differences in dietary behaviors have already appeared during adolescence (Reynolds et al., 1999). For example, adolescent boys eat faster than adolescent girls in Korea (Jeong et al., 2014). Furthermore, compared with boys, girls tend to give consideration to their food choices (Rolls et al., 1991). The incidence of obesity continues to increase worldwide among children and is particularly high in developed countries (Ng et al., 2014). In Japan, while obesity is increasing among boys, leanness is increasing among girls (Ministry of Education, Culture, Sports, Science and Technology, 2018). Adolescent girls’ leanness may lead to future osteoporosis (Rigotti et al., 1991) and delivery of a low-birthweight infant (Ronnenberg et al., 2003). Compared with other countries, Japan shows a specific trend. Thus, it is important to study dietary behaviors in adolescence by gender.

However, few studies have comprehensively assessed adolescent dietary behaviors by gender in Japan. Furthermore, most previous studies were conducted in the U.S. and other Western countries. To address these issues, we conducted a large-scale survey of adolescents’ lifestyles throughout Japan. Based on previous reports (Reynolds et al., 1999, Rolls et al., 1991), we hypothesized that boys tend to adopt unhealthy dietary behaviors to a greater extent than girls. In this study, we aimed to investigate gender differences in unhealthy dietary behaviors among Japanese adolescents.

## 2 Methods

### 2.1 Participants

Of the 10,547 junior and 4807 senior high schools registered in Japan in May 2013, 140 junior (selection rate: 1.3%) and 124 senior (selection rate: 2.6%) high schools were sampled. We used a stratified, single-stage cluster-sampling method to divide Japan into regional blocks and randomly selected schools from each block. To avoid any sampling bias, stratified sampling was performed with regional blocks as the strata. All the students in the sampled schools were the study participants. The sample size was determined by the response rate and confidence intervals based on the variance of the results obtained from a previous study (Osaki and Minowa, 1996).

In the Japanese education system, children enter elementary school at the age of 6 years and leave after 6 years of study. They then enter junior high school for 3 years of study, followed by a further 3 years in senior high school. Education is mandatory in junior high school. In this study, the first to third years of junior high school are called the 7th to 9th grades, and the first to third years of senior high school is called the 10th to 12th grades.

### 2.2 Survey procedure

We sent a letter to the principal of each selected school for cooperation in the survey, along with the same number of questionnaires and envelopes as the number of students enrolled at the school. Schools, where the principals had agreed to the survey and had received questionnaires and envelopes, also required each class teacher to inform the students and ensure the protection of the privacy of the respondents. Teachers explained to the students about confidentiality and voluntary participation, and delivered the completed questionnaires in the sealed envelopes back to our department. The survey was conducted from October 2014 to January 2015. This study was approved by the research members‘ institutional Ethical committee.

### 2.3 Measures

The major sections of the questionnaire were (1) personal data, (2) dietary behaviors, (3) lifestyle, and (4) mental health status. Personal data included gender, school grade, and type of school.

#### 2.3.1 Dietary behaviors

To ensure reliability of the dietary behaviors, we used the questions of the 2007 National Health and Nutrition Survey (NHNS) established by the Ministry of Health, Labor and Welfare (Ikeda et al., 2015). The unhealthy dietary behaviors in the present study were based on definitions used in previous studies (Otsuka et al., 2019, Videon and Manning, 2003, Wijtzes et al., 2015):

1) Skipping breakfast

How often did you eat breakfast during the previous month? (1. Almost every day, 2. Sometimes, 3. Seldom). Skipping breakfast was defined when answered as “Sometimes” or “Seldom.”

2) Snacking

How many times per day did you snack during the previous month? (1. Over 14 times/week, 2. 7–13 times/week, 3. 2–6 times/week, 4. under once/week, 5. None). Snacking was defined when answered as “Over 14 times/week.”

3) Eating out

How many times per day did you eat out during the previous month? (1. Over 14 times/week, 2. 7–13 times/week, 3. 2–6 times/week, 4. under once/week, 5. None). Eating out was defined when answered as “Over 14 times/week” or “7–13 times/week.”

4) Skipping meals

How many times per day did you skip a meal during the previous month? (1. Over 7 times/week, 2. 4–6 times/week, 3. 2–3 times/week, 4. under once/week, 5. None). Skipping meals was defined when answered as “Over 7 times/week” or “4–6 times/week.”

5) Eating alone at dinner

When you ate dinner at home during the previous month, who did you eat with? (1. All the family, 2. Some of the family, 3. Alone, 4. Other). Lack of family dinner was defined when answered as “Alone.”

6) Subjectively poor diet quality

How was the quality of meals at home during the previous month? (1. Very good, 2. Good, 3. Bad, 4, Very bad). Subjectively poor diet quality was defined when answered as “Bad” or “Very bad.”

#### 2.3.2 Covariates

To determine the following covariates, we referred to factors associated with dietary behaviors in previous studies (Fulkerson et al., 2014, Jacka et al., 2010, Kaneita et al., 2006, Kim et al., 2010, Larson et al., 2016, Neumark-Sztainer et al., 1997). The lifestyle questionnaire assessed current smoking, current alcohol drinking, participation in club activities, exercise habits, more than 5 h of internet usage on weekdays, insomnia symptoms, and interest in being healthy. The covariate of internet usage time was derived from a previous study in which Japanese adolescents using ≥5 h of internet on weekdays were categorized as “high-risk” group for problematic internet use (Mihara et al., 2016). Internet addiction in adolescents can negatively affect dietary behaviors (Kim et al., 2010). Insomnia is defined when there is presence of one or more of the following symptoms: difficulty in initiating sleep, difficulty in maintaining sleep, and early morning awakening (Kaneita et al., 2006). Mental health status was assessed using selected items from the 12-item General Health Questionnaire (GHQ-12) (Doi et al., 2003, Goldberg et al., 1976). In this study, we used the assessments for ‘‘depression and anxiety’’ and ‘‘decrease in positive feeling’’ from the GHQ-12. One question from each of the two factors was selected for this study. Students who answered affirmatively to either question were classified as having poor mental health (Suzuki et al., 2011).

### 2.4 Data analysis

First, we calculated the demographic characteristics of the analyzed participants and the 95% confidence intervals. Next, we calculated the prevalence and the 95% confidence interval of each unhealthy dietary behavior by gender and school grade and compared gender differences using the chi-squared test. Finally, to examine the gender differences in unhealthy dietary behaviors, multiple logistic regression analyses were performed. School grade, drinking alcohol, smoking, extracurricular activities, interest in being healthy, exercise habits, insomnia, mental health status, and 5-or-more hours of internet use on weekdays were used as the covariates. We set the level of significance at *P* < 0.01. All analyses were performed using SPSS version 17.0.

## 3 Results

This study enrolled 165,269 students who randomly selected junior and senior high schools. The total number of respondents was 85,931. Overall, 943 questionnaires were excluded because gender was not specified or the responses were inconsistent. Data from the remaining 84,988 questionnaires were analyzed. The effective response rate was 51.4%.

Table 1 shows the demographic characteristics of the participants by gender. The proportion of boys was higher in grades 7–9 and that of girls was higher in grades 10–12. Regarding drinking alcohol, smoking, club activities, and exercise habits, the proportion of boys was significantly higher than that of girls. Regarding poor mental health, interested in being healthy, and heavy Internet users during the weekday, the proportion of girls was significantly higher than that of boys.

**Table 1:** Demographic characteristics of analyzed participants (%).

	Boys N = 41,225	%	95%CI	Girls N = 43,763	%	95%CI
Grade						
Grade 7	5467	13.3	12.9–13.6	5061	11.6	11.3–11.9
Grade 8	5426	13.2	12.8–13.5	5055	11.6	11.3–11.9
Grade 9	5320	12.9	12.6–13.2	5145	11.8	11.5–12.1
Grade 10	9058	22.0	21.6–22.4	9990	22.8	22.4–23.2
Grade 11	8348	20.3	19.9–20.6	9390	21.5	21.1–21.8
Grade 12	7606	18.5	18.1–18.8	9122	20.8	20.5–21.2
Drinking alcohol	3516	8.5	8.3–8.8	3298	7.5	7.3–7.8
Smoking	1101	2.7	2.5–2.8	469	1.1	1.0–1.2
Club activity	28,220	71.5	71.1–72.0	28,469	66.7	66.2–67.1
Excise habit	20,745	52.4	51.9–52.9	13,365	31.2	30.8–31.6
Insomnia	8762	21.3	20.9–21.7	8666	21.0	20.6–21.4
Poor mental health	5002	13.3	13.0–13.6	7500	17.2	16.9–17.6
Interest in being healthy	22,897	55.9	55.4–56.4	26,477	60.8	60.3–61.3
Using the Internet hour >5 h in weekdays	5022	12.4	12.1–12.7	5651	13.1	12.8–13.4

**Fig. 1:**
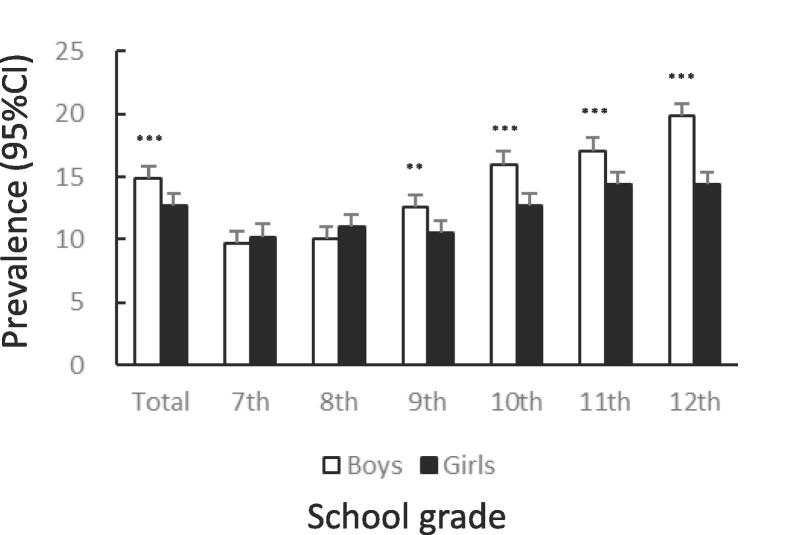

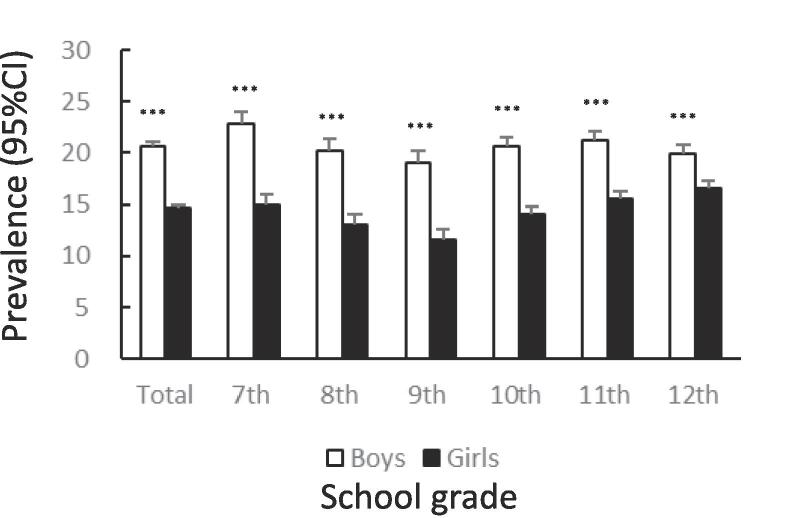

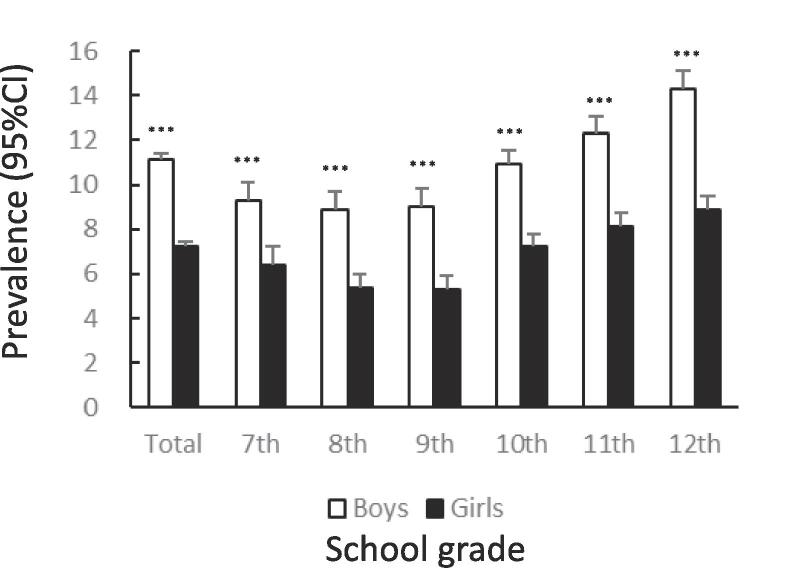

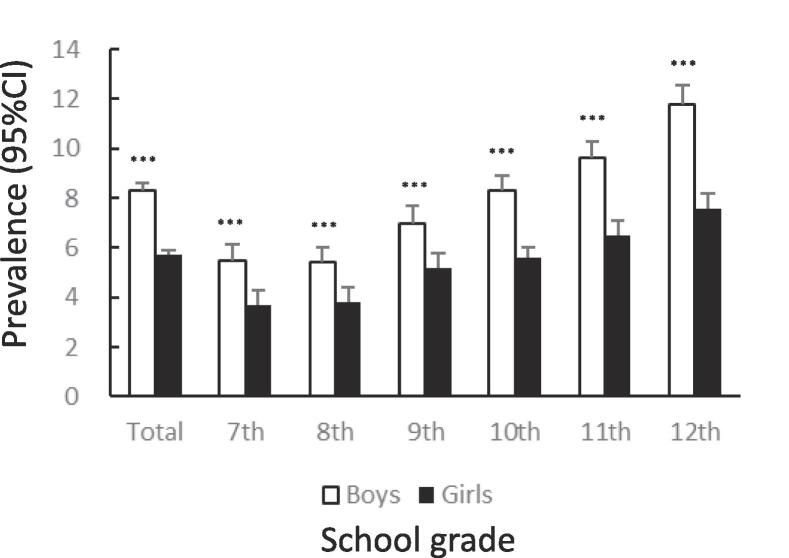

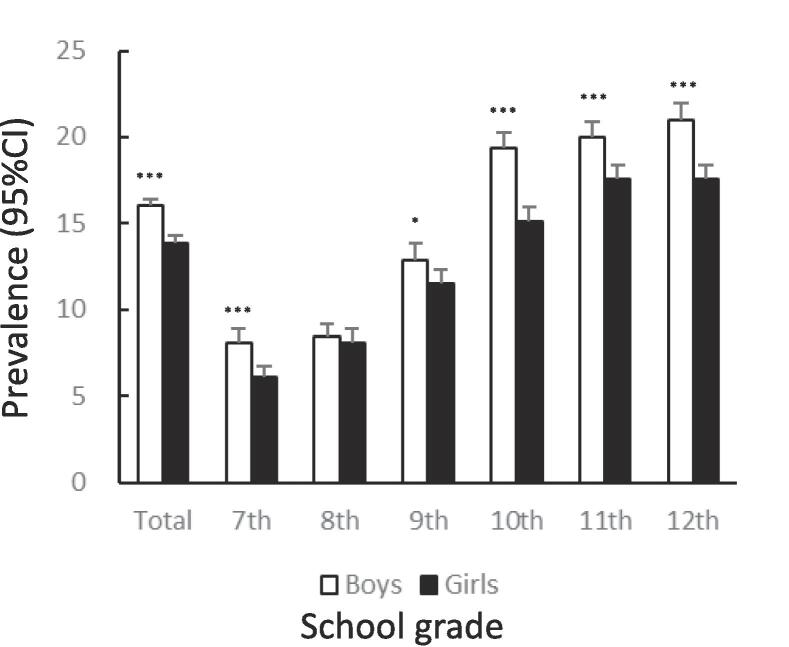

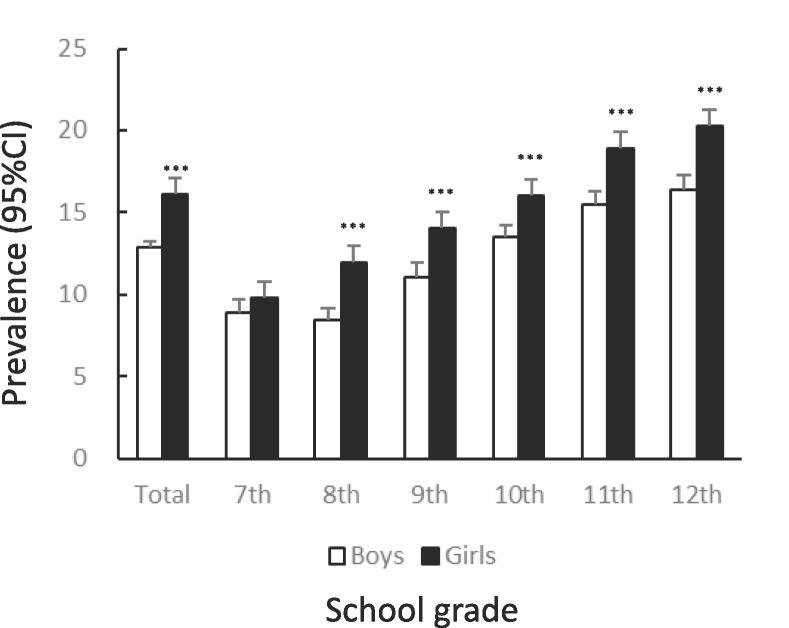
Prevalence of unhealthy dietary behaviors among Japanese adolescents by gender and school grade.

Fig. 1 shows the prevalence of unhealthy dietary behaviors by gender and school grade. The prevalence of skipping breakfast was significantly lower among girls than among boys (12.7%: 95%CI 12.4–13.0 vs. 14.9%: 95%CI 14.6–15.2, χ^2^ = 83.65, p < .001). The tendency to skip breakfast increased among boys and girls with higher grades (Fig. 1A). The prevalence of snacking among girls was significantly lower than that among boys (14.6%: 95%CI 14.3–15.0 vs. 20.6%: 95%CI 20.2–21.0, χ^2^ = 521.4, p < .001). In both boys and girls, this percentage declined after the 7th grade, bottomed out in the 9th grade, and then rose again (Fig. 1B). The prevalence of eating out was significantly lower among girls than among boys (7.2%: 95%CI 6.9–7.4 vs. 11.1%: 95%CI 10.8–11.4, χ^2^ = 372.8, p < .001). In boys and girls, the higher the grade, from 10th to 12th, the higher the prevalence of eating out (Fig. 1C). The prevalence of skipping meals was significantly lower among girls than among boys (5.7%: 95%CI 5.5–6.0 vs. 8.3%: 95%CI 8.0–8.6, χ^2^ = 205.0, p < .001). In boys and girls, the higher the grade in schools, the higher the prevalence of skipping meals (Fig. 1D). The prevalence of eating alone at dinner was significantly lower among girls than among boys (13.9%: 95%CI 13.6–14.3 vs. 16.0%: 95%CI 15.7–16.3, χ^2^ = 73.96, p < .001). The prevalence tended to increase with higher grades in both boys and girls (Fig. 1E). The prevalence of subjectively poor diet quality was significantly higher among girls than among boys (16.1%: 95%CI 15.7–16.5 vs. 12.9%: 95%CI 12.6–13.2, χ^2^ = 175.1, p < .001). The prevalence tended to increase with higher grades in both boys and girls.

Table 2 shows the logistic regression results for the gender differences in unhealthy dietary behaviors among Japanese adolescents. After adjusting for covariates, girls compared to boys were more negatively associated with skipping breakfast [AOR = 0.76 (95% CI 0.73–0.79)], snacking [AOR = 0.67 (95% CI 0.65–0.70)], eating out [AOR = 0.62 (95% CI 0.59–0.66)], skipping meals [AOR = 0.61 (95% CI 0.58–0.65)], and eating alone at dinner [AOR = 0.79 (95% CI 0.76–0.83)]. On the contrary, girls were more positively associated with subjectively poor diet quality [AOR = 1.19 (95% CI 1.14.1.24)].

**Table 2:** Logistic regression results for the gender differences about unhealthy dietary behaviors among Japanese adolescents.

	Girls compared with boys					
Unhealthy dietary behaviors	Crude OR	95%CI	p-value	Adjusted OR	95%CI	p-value
Skipping breakfast	0.83	0.80–0.86	<0.001	0.76	0.73–0.79	<0.001
Snacking	0.66	0.63–0.68	<0.001	0.67	0.65–0.70	<0.001
Eating out	0.62	0.59–0.65	<0.001	0.62	0.59–0.66	<0.001
Skipping meals	0.67	0.64–0.71	<0.001	0.61	0.58–0.65	<0.001
Eating alone at dinner	0.85	0.81–0.88	<0.001	0.79	0.76–0.83	<0.001
Subjectively poor diet quality	1.30	1.25–1.35	<0.001	1.19	1.14–1.24	<0.001

Participants with missing data were excluded from the analysis.

Adjusted for grade, club activity, exercising habit, mental health, long time internet user, alcohol drinking, smoking, and interest in being healthy at multiple logistic regression.

P value was calculated using the multiple logistic regression analysis.

“Skipping Breakfast” was calculated as the sum of “Seldom and Sometimes”. “Snacking” was defined as “Over 14 times/week”. Eating out was defined as “Over 14 times/week and 7–13 times/week”. Skipping meals was defined as the sum of percentage “Over 7 times/week and 4–6 times/week”. Eating dinner at alone was defined as “Alone”. Subjectively poor diet quality was defined as the sum of “Very bad and Bad”.

AOR: adjusted odds ratio; CI: confidence interval;

## 4 Discussion

This is the first study to examine gender differences in unhealthy dietary behaviors among Japanese adolescents. The results suggest that gender differences in dietary behaviors appear to be partly related to their beliefs regarding healthy eating. This study had two strengths: 1) a nationwide survey and 2) an extremely large sample. Many studies have examined a few unhealthy dietary behaviors, such as skipping breakfast. However, we found almost no similar studies examining the prevalence of a variety of dietary behaviors. Furthermore, few studies have been conducted on adolescents’ dietary behaviors in Asia. Therefore, this study makes an important contribution to health education.

Overall, the present study indicated that girls tended to adopt regular dietary behaviors as compared to boys, but they seemed dissatisfied with the quality of their diet. This is partially consistent with previous studies (Reynolds et al., 1999, Rolls et al., 1991). Reasons for different dietary behaviors can be found in nutrition knowledge and attitude. Girls have more nutrition knowledge than boys and are extremely self-conscious about their bodies (Pirouznia, 2001, Rampersaud et al., 2005). In contrast, girls showed lower intake of essential vitamins and minerals and lesser ingestion fruits and vegetables as compared to boys (Johnson et al., 1994). Boys tended to have meals that were higher in total fat and saturated fat as compared to girls (Munoz et al., 1997). Thus, boys and girls showed a significant difference in their dietary behaviors. In future studies, it is necessary to identify these differences.

Previous studies on skipping breakfast and meals have shown inconsistent gender differences (Oba et al., 2016, Smetanina et al., 2015, Smith et al., 2017, Vereecken et al., 2009). In Western countries, girls skip breakfast and other meals more frequently than boys (Smith et al., 2017, Vereecken et al., 2009). However, similar to the present study, another cross-sectional study among Japanese adolescents reported that there is a significant association between skipping meals and male gender (Oba et al. 2016). Although the reason for this gender difference is not known, more boys reported frequent snacking and eating out as compared to girls, and the gender distribution could explain it. Despite the low frequency of skipping meals among adolescent girls, the prevalence of lean women in their 20 s is very high (21.7%) as compared to lean men (9.1%) (Ministry of Health, Labour and Welfare, 2017). Thus, it is suggested that Japanese young girls are more worried about their weight than the boys are. Girls are known to present more body image distortion as compared to boys (Smolak, 2004). Moreover, girls are more likely to sense themselves as overweight when in fact they are average or even underweight, while many boys are likely to be concerned about being overweight when they are actually obese. Skipping meals is often used as a weight-loss method (Neumark-Sztainer et al., 2012). The present study showed that Japanese girls might not skip meals, but instead reduce the amount of food and worsen the quality of their food, for e.g., cut down intake of carbohydrates. In future studies, the question of how they feel about their own weight and the nutrition or energy from meals should be addressed.

We found significant differences in the prevalence of snacking by gender. Adolescent boys snacked more often perhaps because adolescent boys have voracious appetites, which might result in hunger and the need to eat during break times while undergoing club activities. Interestingly, the gap between boys and girls with respect to snacking diminished as the grade increased. A cross-sectional study in Poland youth reported that women snack more frequently on sweets, biscuits, nuts, and seeds, whereas men tend to snack on salty snacks, add sugar to beverages, and add salt to dishes (Zaborowicz et al., 2016). Participants with insufficient nutritional knowledge snack more frequently on salty snacks rather than fruit (Zaborowicz et al., 2016). Thus, age and gender may affect snacking behaviors. Snacking is generally considered a factor in the development of overweight and obesity in childhood (Nicklas et al., 2003). These results suggest the necessity of a multi-dimensional survey with respect to adolescents’ age and gender for healthy snacking behavior.

Regarding the frequency of family dinners, consistent with previous studies, we found that boys ate alone at dinner less often than girls (Goldfield et al., 2011, Shirasawa et al., 2018). One explanation for this is that boys often eat fast food after school and club activities; thus, they have fewer opportunities for family dinners. Previous studies have reported that family meal times may act as a protective factor against nutritional health-related problems encountered during adolescence, including unhealthy dietary behaviors (Hammons and Fiese, 2011). Another explanation is that the schedule of the cram school may affect family dinners. Because of adolescents’ cram school sessions and their parents’ jobs, the number of times a Japanese family eats a meal together has reduced (Kojima, 2011). Therefore, our findings suggest that health educators need to promote the importance of family dinners and the risks related to eating alone.

In contrast to the above results, we found that the prevalence of subjectively poor diet quality was higher among girls. Studies have reported that girls tend to hold stronger diet-related beliefs than boys (Davy et al., 2006, Wardle et al., 2004). Thus, girls may underestimate the quality of their diet, while boys may overestimate them. Although girls regularly eat meals, the result also suggests that girls may be restricted to the amount of meals. Future research is needed to use objective indicators such as the Diet Quality Index (Vyncke et al., 2013).

The results of the current study also suggested that some dietary behaviors such as skipping breakfast, eating out, eating alone, subjective poor diet quality were likely to have an interaction between gender and school grade which meant age. The findings in the current study support in line with the several previous studies which show that pubertal status may affect on skipping meals and snacks (Lee and Lee, 2013, Nu et al., 1996). Another explanation for that is that high school students (grade10-12) tend to eat in accordance with their own daily rhythms, thus dietary behaviors are easily disturbed. Therefore, our findings suggest that health educators need to change the content of education in dietary behaviors depending on the grade

This study had several limitations. First because it was a cross-sectional study, the cause and effect relationships for each dietary behavior, or how these may have changed over time, could not be determined. For example, poor mental health status could affect dietary and other behaviors more when compared to healthy mental status (Brausch and Gutierrez, 2009). In the present study, we could use only two items from GHQ-12 for mental health due to limited space in the questionnaire. To focus more on the adolescents’ mental health status in future surveys, all GHQ items may need to be included for accurate measurement. Second, data for the study were collected via a self-administered questionnaire. Using a validated method to assess meal balance over the previous 24 h, for example, would have provided strong evidence that could have been used in the study. Third, no data were obtained on participants’ weights or socio-economic factors, such as family income or the educational levels of the participants’ parents. Future research should include socio-economic factors and their actual and ideal weights. Fourth, about 40% of non-responses existed, because minors aged <20 years in Japan are prohibited by law from smoking and drinking alcohol. Therefore, schools and students tend to be non-cooperative in responding to questions on their smoking and drinking status. However, the present survey response rate had some acceptability (Johnson and Wislar, 2012).

## 5 Conclusion

The current study found that dietary behaviors differed between genders, and these differences varied among junior and senior high school. Future research should examine factors in the home environment, such as socioeconomic status (SES) and use of electronic devices during meals. Our results highlight the importance of developing gender-specific prevention strategies. Integrating strategies in health education in schools could contribute to improving adolescent health. Interventions should focus on healthy eating behaviors and improving food quality. It would be advisable to focus on developing healthy eating habits in boys, and education in girls regarding the necessary nutrients and proper energy intake. Schools need to not only teach the importance of proper dietary behaviors, but also support modeling and reinforcing healthy dietary behaviors. Adolescents have easy access to high fat and high sugar food and beverage products around school. Hence, schools need to create a supportive and integrated nutrition environment.

## Funding

This study was supported by a Health Science Research Grant from the Ministry of Health, Labor, and Welfare of the Japanese Government.

## CRediT authorship contribution statement

**Yuichiro Otsuka:** Formal analysis, Data curation, Writing - original draft. **Yoshitaka Kaneita:** Project administration, Writing - review & editing. **Osamu Itani:** Software, Supervision, Visualization. **Maki Jike:** Investigation, Methodology. **Yoneatsu Osaki:** Formal analysis, Funding acquisition. **Susumu Higuchi:** Project administration, Resources. **Hideyuki Kanda:** Conceptualization, Methodology.

## Declaration of Competing Interest

The authors declare that they have no known competing financial interests or personal relationships that could have appeared to influence the work reported in this paper.
